# Efgartigimod treatment for generalized myasthenia gravis: a single-center case series of 16 patients

**DOI:** 10.3389/fneur.2024.1472845

**Published:** 2024-10-14

**Authors:** Toshiya Nomura, Michie Imamura, Masao Imura, Hironori Mizutani, Mitsuharu Ueda

**Affiliations:** Department of Neurology, Graduate School of Medical Sciences, Kumamoto University, Kumamoto, Japan

**Keywords:** myasthenia gravis, autoimmune diseases, efgartigimod alfa, neonatal Fc receptor, case series

## Abstract

**Background:**

Efgartigimod was approved in Japan in January 2022 for the treatment of generalized myasthenia gravis (gMG), regardless of antibody status. This case series describes a real-world experience in Japan of efgartigimod treatment for gMG patients with diverse backgrounds.

**Methods:**

We retrospectively analyzed the medical records of 16 Japanese patients (11 females and five males, mean age 40.4 years) with gMG who received efgartigimod at the Kumamoto University Hospital between August 2022 and September 2023. The outcomes were Quantitative Myasthenia Gravis (QMG) responders (≥ 3 point reduction), IgG levels, and change in prednisolone dose, in the first cycle of efgartigimod.

**Results:**

Fifteen patients completed one cycle of efgartigimod. Of the 14 patients for whom QMG scores were obtained, 10 patients were QMG responders. Four of the five patients with Myasthenia Gravis Foundation of America class V were QMG responders. Improvement in QMG after efgartigimod treatment was observed in one patient with myasthenic crisis and in one refractory patient who had unsuccessful eculizumab treatment. The mean reductions from baseline in IgG levels at weeks 1, 2, 3, and follow-up were 38.3, 56.1, 63.1, and 43.9%, respectively. A decrease in prednisolone dose was observed in seven patients. The most common adverse events were headache (three patients) and diarrhea (two patients). One patient discontinued efgartigimod treatment due to a treatment-related adverse event of rash.

**Conclusion:**

Improvements in the outcomes of patients with gMG, including patients with severe gMG, myasthenic crisis, and refractory to anti-complementary therapy, were observed after the first cycle of efgartigimod treatment. Our real-world experience in Japan suggests the future possibilities for the treatment with efgartigimod for gMG with diverse backgrounds.

## 1 Introduction

Myasthenia gravis (MG) is an acquired neuromuscular junction disorder caused by autoantibodies to the postsynaptic membrane, including the nicotinic acetylcholine receptor (AChR) and muscle-specific tyrosine kinase. Meta-analyses estimate the incidence rate and prevalence rate of MG are from 5 to 30 cases per million person years and between 10 and 20 cases per 100,000 general population, respectively. MG shows a first peak around 30 years of age, predominantly affecting females, and a second peak after 50 years, with a higher prevalence in males (1). In most patients, immunotherapy includes corticosteroids and immunosuppressive drugs to control symptoms (2, 3). However, a subset of patients with generalized myasthenia gravis (gMG) is refractory to these treatments, has intolerable side effects, and/or requires rescue treatments such as intravenous methylprednisolone, intravenous immunoglobulin, or plasmapheresis (4). Monoclonal antibodies, such as eculizumab and ravulizumab, which specifically target the terminal C5 component, improve symptoms in refractory gMG; however, these treatments are only approved for AChR antibody-positive gMG, and some patients have limited or no clinical improvement while receiving them (5).

Neonatal Fc receptor antagonists reduce the recycling of IgG, resulting in a reduction in pathogenic antibodies and clinical improvement of gMG. Efgartigimod is a first-in-class neonatal Fc receptor antagonist. In a phase 3 randomized controlled trial (ADAPT trial), 68% of patients receiving efgartigimod treatment showed improvement in Myasthenia Gravis Activities of Daily Living (MG-ADL) compared with 30% of patients receiving placebo (6). Based on the results of clinical trials (6–8), efgartigimod was first approved in the United States in December 2021 for the treatment of gMG in adults who are AChR antibody-positive (9), and in the EU in August 2022 as an add-on treatment to standard therapy (10). In Japan, efgartigimod was approved for the treatment of gMG regardless of the antibody status in January 2022 and was launched in May of the same year (11). Following the approval of efgartigimod, observational studies based on the real-world experiences with this new treatment for gMG have been reported in different countries (12–17). In addition, the role of efgartigimod in treating conditions beyond MG has been reported (18). However, there are limited reports on the experience and outcomes of efgartigimod treatment in clinical practice in gMG with various backgrounds, including age, type or severity of MG, and antibody status. Moreover, according to strict enrolment criteria, the ADAPT trial excluded certain populations such as patients with aged <18 years, patients who had experienced a myasthenic crisis (MGFA class V) and patients who had received eculizumab or rituximab within the 6 months of screening.

In this case series, we describe our clinical experience with efgartigimod treatment at a single center in Japan for patients with gMG, including patients with different backgrounds from those in the ADAPT trial (6).

## 2 Materials and methods

We retrospectively analyzed the medical records of 16 patients with gMG at the Kumamoto University Hospital in Kumamoto, Japan. This study was approved by the Human Ethics Review Committee of Kumamoto University (No. 2854) and was conducted in accordance with the Declaration of Helsinki. Due to the retrospective nature of this study using anonymized data from medical records, patient consent was waived, and opt-out consent, in which participants were provided information about this study and enrolled unless they actively opted out, was applied. Additional consent for publication was obtained from the individual participants, whose detailed information as the “case presentations” is included in this article.

The diagnosis of gMG was made by neurologists according to the diagnostic criteria of the Japanese guidelines (19). Briefly, the “definite” diagnosis of MG made if either of the following is true (17): (i) One or more items from “A) symptoms” is true and any item of “B) pathogenic autoantibodies” is true; (ii) One or more items from “A) symptoms” is true and any item of “C) neuromuscular junction disorders” is true and other diseases can be ruled out. “A) symptoms” include (1) blepharoptosis, (2) eye movement disorder, (3) facial muscle weakness, (4) dysarthria, (5) dysphagia, (6) mastication disorder, (7) cervical muscle weakness, (8) limb muscle weakness, and (9) respiratory disorders. “B) pathogenic autoantibodies” are (1) anti-acetylcholine receptor antibody-positive and (2) anti-muscle-specific receptor tyrosine kinase antibody-positive. “C) neuromuscular junction disorders” consisted of (1) positive eyelid easy fatigability test, (2) positive ice pack test, (3) positive edrophonium chloride (Tensilon) test, (4) positive on repetitive stimulation test, and (5) jitter increase on single-fiber electromyography test.

Inclusion criteria were as follows: patients with gMG with age ≥ 15 years old, received at least one dose of efgartigimod at the Kumamoto university hospital between August 2022 and September 2023. According to the package insert (11), efgartigimod (10 mg/kg) was administered intravenously over 1 h once weekly for 4 weeks in one cycle, and each cycle was repeated thereafter.

The following data were collected from medical records: age, sex, disease duration and subtype of MG, antibody positivity, Myasthenia Gravis Foundation of America (MGFA) classification (20), MG-ADL score (21), Quantitative Myasthenia Gravis (QMG) score (22), history of thymectomy, prior maintenance treatment for MG, and rescue treatment for MG during the previous 1 year. The clinical outcomes of this study were the proportion of patients who were QMG responders in the first cycle, total IgG levels during the first cycle, and the change in prednisolone dose from baseline to follow-up. QMG responders were defined as patients who had a clinically meaningful improvement (≥ 3 point reductions) (22) from baseline to 4 weeks (after the first cycle of the efgartigimod treatment). The follow-up visit for the prednisolone dose was defined as the last visit when the patients underwent a medical examination between August 2022 and September 2023. The follow-up visit for IgG levels was defined as the first visit when IgG levels were measured after week three. We also collected data on any reported adverse events from the medical records. During this study, adverse events were monitored by vital signs, physical examination and laboratory assessments. For case presentations of patients whose clinical course was considered important, we collected QMG scores at the visit from the time of referral to our hospital and to the last visit, as well as treatment information for MG between the diagnosis and the last visit. Descriptive analysis was performed for baseline patient characteristics and clinical outcomes, with number and proportion (%) for categorical data and mean with standard deviation (SD) for continuous data. All statistical analyses were performed using Microsoft Excel 2021 (Microsoft Corporation, Redmond, WA, United States).

## 3 Results

### 3.1 Baseline characteristics

Eleven female and five male patients with gMG who received efgartigimod between August 2022 and September 2023 were included in this study. All 16 patients were Japanese of Asian ethnicity. The mean (SD) age was 40.4 (13.2) years (Table 1). One patient (Patient No. 3) was aged 15. The mean (SD) disease duration was 14.3 (11.6) years. The disease subtypes were: 12 patients of early onset MG, one patient of late-onset MG, two patients of thymoma-associated MG, and one patient of muscle-specific tyrosine kinase MG. Eleven of the 16 patients had severe gMG with an MGFA classification of III or higher, including five patients with an MGFA classification of V. The mean baseline QMG and Myasthenia MG-ADL score were 13.8 and 6.6, respectively. Twelve patients underwent a thymectomy. Prior to the initiation of efgartigimod treatment, all 16 patients were taking corticosteroids. Of these, 15 patients were taking immunosuppressive drugs such as tacrolimus and cyclosporine, and one patient each had received eculizumab or rituximab. Thirteen patients had received rescue treatments including intravenous immunoglobulin or plasma exchange during the previous 1 year.

**Table 1:** Demographics and clinical characteristics of 16 patients treated with efgartigimod for generalized myasthenia gravis.

Patient No.	Sex	Age (years)	Age at gMG onset (years)	Duration of gMG (years)	Type/Antibody	MGFA class	QMG score	MG-ADL score	Thymectomy	Prior maintenance treatments	Rescue treatment during previous 1 years
1	F	34	30	4	EOMG/Anti-AChR	V	24	12	Yes	PSL 10 mg	Plasma exchange
										Tacrolimus 3 mg	
2	F	31	13	18	EOMG/Anti-AChR	V	21	11	Yes	PSL 10 mg	IVIg 1 course
										Tacrolimus 3 mg	
3	F	15	10	5	EOMG/Anti-AChR	IIIa	19	6	Yes	PSL 18 mg	IVIg 2 course
										Tacrolimus 3 mg	
4	F	57	14	43	EOMG/Anti-AChR	IVa	18	3	Yes	PSL 20 mg (alternative day)	IVIg 1 course
										Cyclosporin 170 mg	
										Eculizumab	
5	F	39	25	14	EOMG/Anti-AChR	IIa	18	11	Yes	PSL 15 mg	IVIg 2 course
										Tacrolimus 3 mg	
6	M	39	36	3	EOMG/Anti-AChR	IIa	15	5	Yes	PSL 20 mg	IVIg 2 course
										Tacrolimus 3 mg	
7	F	36	30	6	EOMG/Anti-AChR	V	14	5	Yes	PSL 10 mg	IVIg 1 course
										Tacrolimus 3 mg	
8	F	58	25	33	TAMG/Anti-AChR	IIIa	13	6	Yes	PSL 25 mg	-
										Tacrolimus 3 mg	
										Rituximab	
9	M	40	33	7	EOMG/Anti-AChR	V	13	8	Yes	PSL 11 mg	-
										Cyclosporin 200 mg	
10	M	50	44	6	EOMG/Anti-AChR	IIa	11	7	No	PSL 30 mg	IVIg 2 course
										Tacrolimus 3 mg	
11	M	30	18	12	TAMG/Anti-AChR	V	9	2	Yes	PSL 9 mg	IVIg 1 course
										Cyclosporin 200 mg	
12	F	32	26	6	EOMG/Anti-AChR	IIIb	8	5	No	PSL 15 mg	-
13	F	19	2	17	EOMG/Anti-MuSK	IIIa	7	1	No	PSL 20 mg	IVIg 1 course
										Tacrolimus 3 mg	
14	F	52	29	23	EOMG/Anti-AChR	IIa	6	5	Yes	PSL 6 mg	IVIg 1 course
										Tacrolimus 3 mg	
15	M	59	56	3	LOMG/Anti-AChR	IIa	8	6	No	PSL 30 mg	IVIg 2 course
										Tacrolimus 3 mg	
16	F	55	27	28	EOMG/Anti-AChR	IIIa	17	13	Yes	PSL 20 mg	IVIg 2 course
										Cyclosporin 240 mg	

F, Female; M, Male; gMG, Generalized myasthenia gravis; EOMG, Early onset myasthenia gravis; Anti-AChR, Anti-acetylcholine receptor; TAMG, Thymoma-associated myasthenia gravis; Anti-MuSK, Anti-muscle-specific tyrosine kinase; LOMG, Late-onset myasthenia gravis; MGFA, Myasthenia Gravis Foundation of America; QMG, Quantitative myasthenia gravis; MG-ADL, Myasthenia gravis activities of daily living; PSL, Prednisolone; and IVIg, Intravenous immunoglobulin.

### 3.2 Treatment

Of the 16 patients who started efgartigimod treatment, 15 patients completed one cycle of efgartigimod. One patient (Patient No. 15) discontinued efgartigimod treatment after the second infusion of the first cycle due to adverse event of rash (Malassezia folliculitis). Twelve patients received a second cycle of efgartigimod; for these patients, the mean (SD) interval between the first and the second cycle was 13.6 (8.9) weeks. Seven patients received a third cycle, four patients received a fourth cycle, and two patients received a fifth cycle.

### 3.3 Quantitative myasthenia gravis score

**Figure 1:**
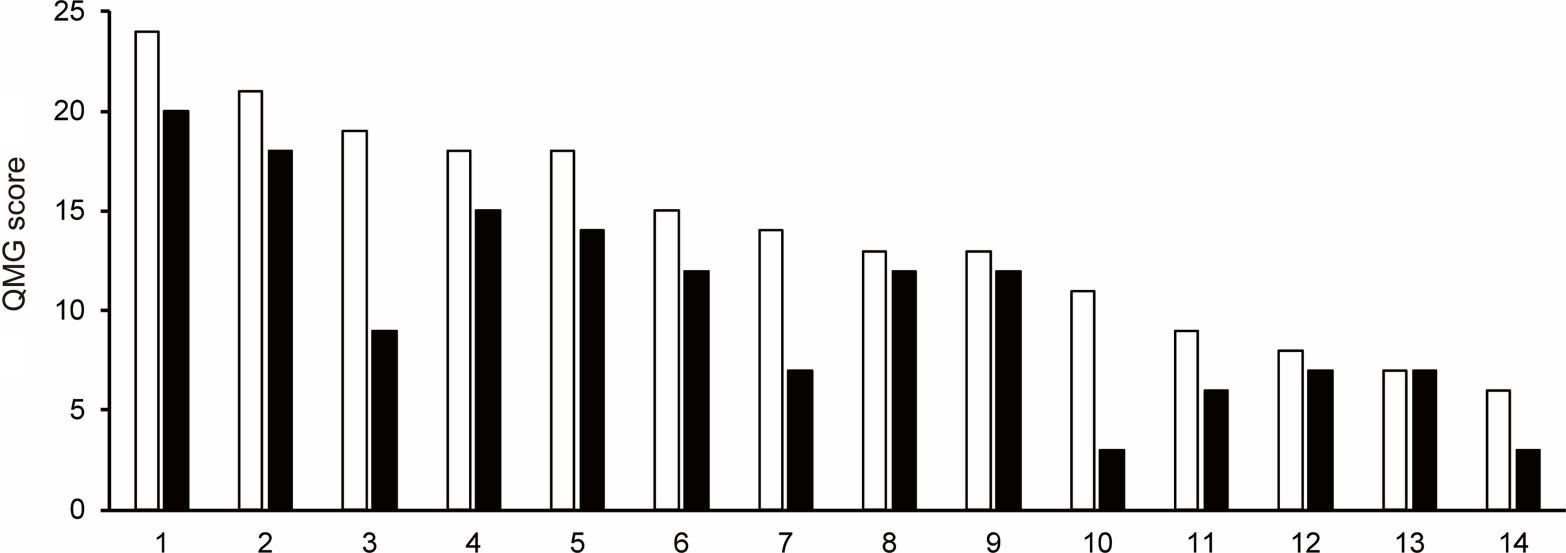
The change in Quantitative Myasthenia Gravis score for each patient from baseline (white bar) to after the first cycle of the efgartigimod treatment (black bar). Each number on the *X*-axis indicates a Patient No. 10 of 14 patients (71.4%) were QMG responders (≥3 point reduction the QMG score) in the first treatment cycle.

The QMG score was not recorded after the first cycle of efgartigimod treatment in one patient (Patient No. 16) of 15 patients those completed one cycle of efgartigimod. Of the remaining 14 patients whose QMG scores were analyzed, the QMG score decreased in 13 patients (Patient No. 1–12 and 14) and remained unchanged in one patient (Patient No. 13) after completing the first cycle of treatment with efgartigimod (Figure 1). Ten of 14 patients (71.4%) were QMG responders (≥ 3 point reduction the QMG score) in the first treatment cycle. Four (Patient No. 1, 2, 7, and 11) of the five patients with MGFA class V were QMG responders during the first treatment cycle.

### 3.4 IgG levels

**Figure 2:**
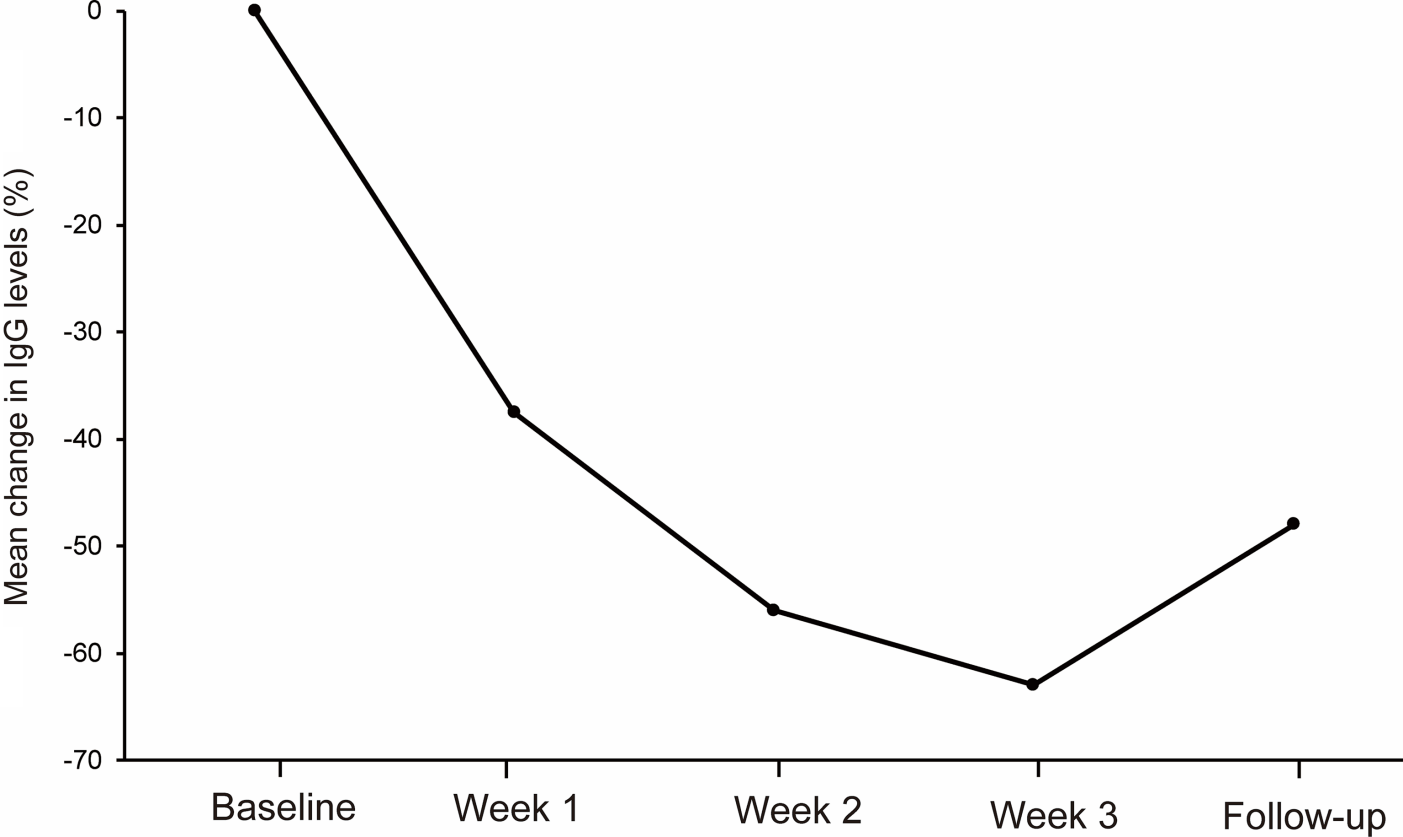
The reductions in total IgG levels from baseline at week 1, week 2, week 3 and follow-up during the first cycle of the efgartigimod treatment. The mean (SD) percentage reductions in IgG levels from baseline at weeks 1, 2, and 3, and follow-up were 38.3 (6.2)%, 56.1 (6.8)%, 63.1 (5.8)%, and 43.9 (20.4)%, respectively.

The mean IgG levels decreased compared to the baseline IgG levels during the first cycle of efgartigimod (Figure 2). The mean (SD) percentage reductions in IgG levels from baseline at weeks 1, 2, 3, and follow-up were 38.3 (6.2)%, 56.1 (6.8)%, 63.1 (5.8)%, and 43.9 (20.4)%, respectively. The maximum mean percentage reductions in IgG levels from baseline was observed at week 3 of the first cycle or before the fourth efgartigimod infusion.

### 3.5 The change from baseline to follow-up in prednisolone dose

**Figure 3:**
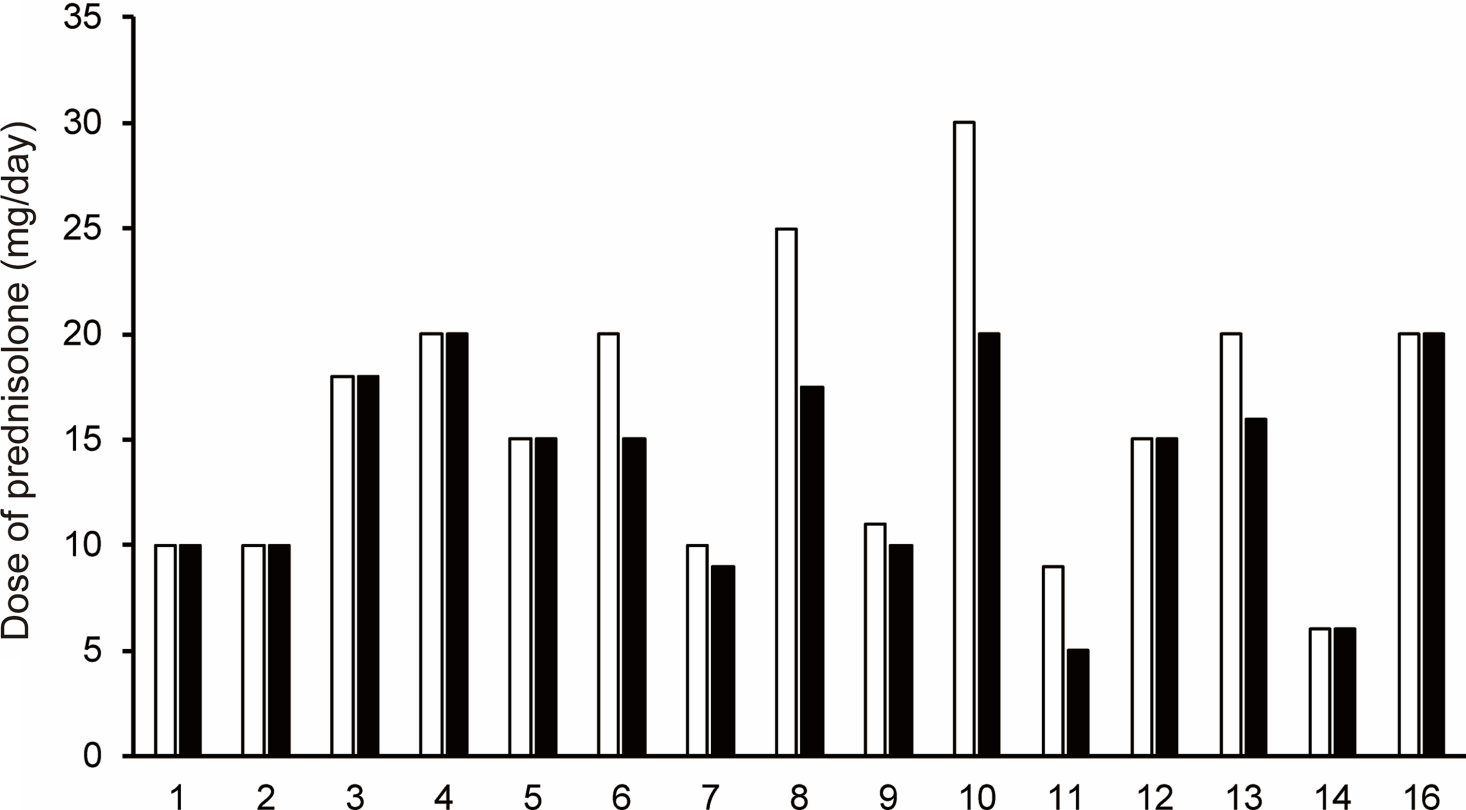
The change in prednisolone dose for each patient from baseline (white bar) to the follow-up after the first cycle of efgartigimod treatment (black bar). Each number on the X-axis indicates a Patient No. At follow-up, the prednisolone dose was decreased compared to the baseline prednisolone dose in seven of 15 patients who were treated with efgartigimod (Patients No. 6–11, and 13).

At follow-up, the prednisolone dose was decreased compared to the baseline prednisolone dose in seven of 15 patients who were treated with efgartigimod (Patients No. 6–11, and 13; Figure 3). Mean prednisolone dose in baseline and follow-up was 15.9 mg/day and 13.8 mg/day, respectively; hence, mean prednisolone dose reductions was 2.1 mg/day. The maximum change from baseline to follow-up in the prednisolone dose, which was 10 mg/day, was observed in patient No 10. after 2 cycles of efgartigimod at an interval of 36 weeks.

### 3.6 Adverse events

The most frequently reported adverse events were headache (three patients, 18.8%) and diarrhea (two patients, 12.5%). No fatal or serious adverse event were observed during the study. An adverse event leading to treatment discontinuation was a rash in one of the 16 patients, which was judged to be related to the administration of efgartigimod. The patient had a history of Malassezia folliculitis. The rash worsened after two infusions in the first cycle of efgartigimod. The patient was treated successfully for the rash, and efgartigimod treatment was discontinued.

### 3.7 Case presentations of two patients

Of the 16 patients in the study, we present case reports of two patients whose clinical course of treatment with efgartigimod for gMG was considered to have educational implications. Patient No. 4 was the only case to be switched from eculizumab to efgartigimod treatment, and Patient No. 7 was the case whose QMG score improved the most by the first cycle of efgartigimod treatment among the patients who had experienced myasthenic crises before the initiation of efgartigimod treatment.

**Figure 4:**
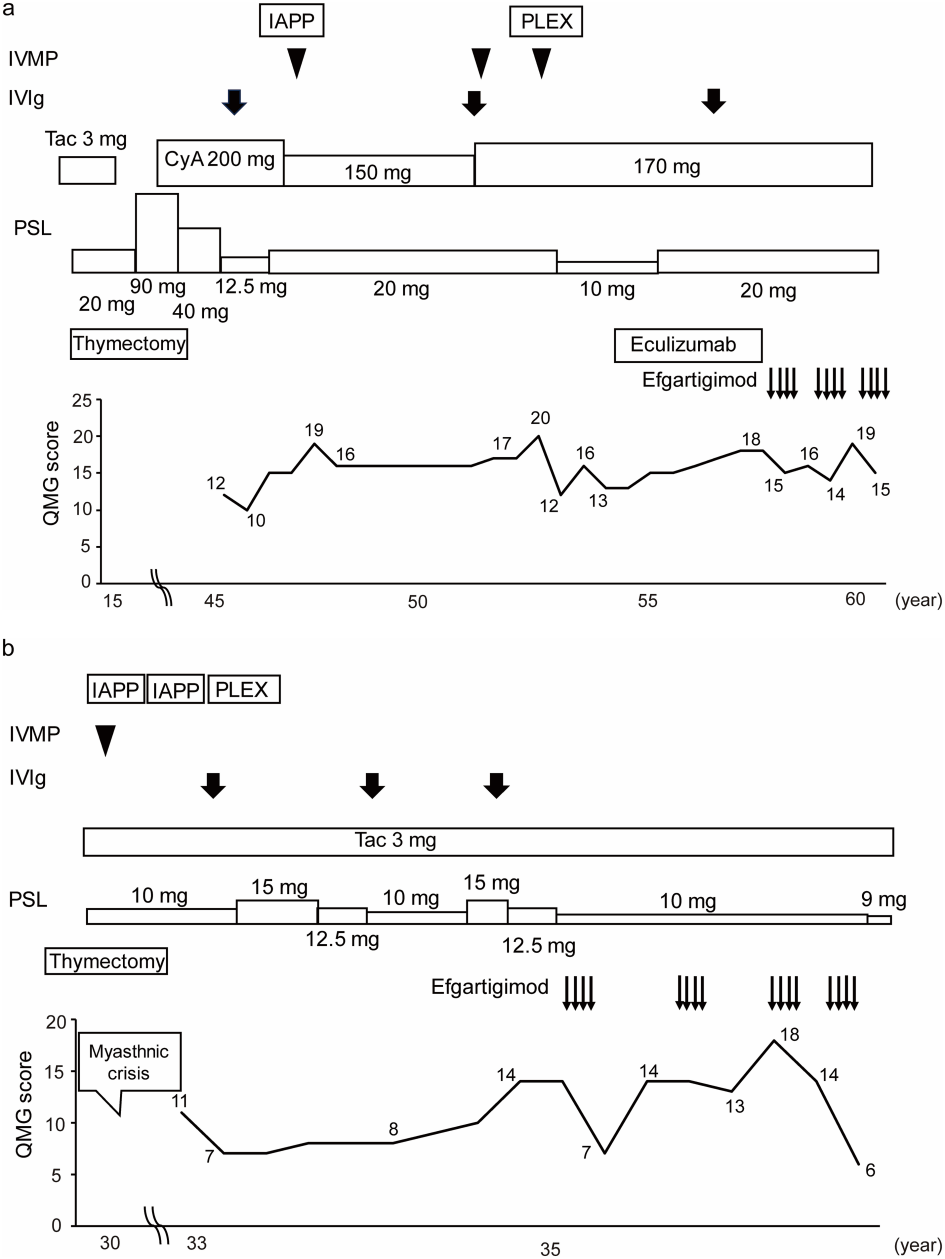
Case presentations of **(a)** Patient No. 4 and **(b)** Patient No. 7. Of the 16 study patients, Patient No. 4 was the only case to be switched from eculizumab to efgartigimod treatment, and Patient No. 7 was the case whose QMG score was improved the most by the first cycle of efgartigimod treatment among the patients who had experienced myasthenic crises. IVMP, Intravenous methylprednisolone; IVIg, Intravenous immunoglobulin; IAPP, Immunoadsorption plasmapheresis; PLEX, Plasma exchange; Tac, Tacrolimus; CyA, Cyclosporine; PSL, Prednisolone; QMG, Quantitative myasthenia gravis; yo, Years old.

Patient No. 4 (Figure 4a) was a 57-year-old female with a 43-year history of MG. She was referred to our hospital at the age of 46 years. She required oral prednisolone and cyclosporine for maintenance treatment, and intravenous methylprednisolone, intravenous immunoglobulin, immunoadsorption plasmapheresis, and plasma exchange for rescue treatment. Eculizumab treatment was initiated along with prednisolone and cyclosporine at the age of 53 years. She was treated with eculizumab for approximately 4 years, but her symptoms gradually worsened, and the prednisolone dosage was increased. Efgartigimod treatment was initiated at 57 years of age. After three cycles of efgartigimod treatment, the patient’s symptoms gradually improved. The QMG score was 15 points at the last visit, which was improved from her pre-treatment score of 18 points.

Patient No. 7 (Figure 4b) was a 36-year-old female with a 6-year history of MG. The patient underwent a thymectomy at the age of 30 years. After surgery, she experienced myasthenic crisis and was treated with immunoadsorption plasmapheresis and intravenous methylprednisolone. She was referred to our hospital at the age of 33 years. She required oral prednisolone and tacrolimus for the maintenance treatment, and intravenous immunoglobulin for rescue treatment. Efgartigimod treatment was initiated with the additional 10 mg/day dose prednisolone and tacrolimus. After four cycles of efgartigimod treatment, the patient’s symptoms gradually improved. The QMG score, which was 14 points at pretreatment, was seven points after the first cycle of efgartigimod, and six points at the last visit. In addition, the prednisolone dose was reduced to 9 mg/day. Although the patient may have needed to continue repeated doses of efgartigimod, this treatment was available on an outpatient basis, allowing her to work part-time.

## 4 Discussion

In this study, we described the outcomes of efgartigimod treatment for gMG in clinical practice through a retrospective analysis of consecutive patients at our institution. We also present data on efgartigimod treatment in populations that were not evaluated in the ADAPT trial (6). Efgartigimod is a relatively new therapeutic option with the potential to fill an unmet need in the treatment strategy for gMG owing to its unique mechanism of action of neonatal Fc receptor inhibition. The clinical significance of efgartigimod for the treatment for gMG was investigated in the ADAPT trial (6), which demonstrated its efficacy and tolerability. In general, a potential limitation of clinical trials is that study results are obtained in settings strictly defined by protocols, including the fact that certain populations (e.g., critically ill patients) may be excluded from the study according to enrolment criteria. Therefore, obtaining data from patients with diverse background characteristics in a real-world setting is a post-marketing issue (12–17).

The proportion of QMG responders, an outcome related to the efficacy of efgartigimod treatment in this study, was consistent with the results of the secondary endpoints of the ADAPT trial (6). In this study, 71% (10 of 14) of patients showed clinically meaningful improvements in QMG scores after the first cycle. In the ADAPT trial, 63% (41 of 65) of the patients in the efgartigimod group were QMG responders in the first cycle (6). Overall, the patients in this cohort were similar in sex, antibody status, and history of thymectomy to those in the ADAPT trial. The ADAPT trial included patients from different races, such as White, Black or African American, and Asian, but this study included only Japanese or Asian patients. A large population of patients in this study had severe gMG with an MGFA classification of III or higher compared to those in the ADAPT trial. In addition, more patients in this study were treated with corticosteroids and/or immunosuppressive drugs than in the ADAPT trial. Unlike this study and the clinical trial, in previous studies (12–17), the proportion of QMG responders was not evaluated as an outcome related to the efficacy of efgartigimod treatment. On the other hand, the proportion of MG-ADL responders, or the primary endpoint of the ADAPT trial, was evaluated in the majority of the previous studies (12–17). QMG score, which is not a simple evaluation method, can objectively assess the severity of MG. Therefore, the findings of this study highlight the efficacy of efgartigimod treatment and its objectiveness.

We emphasize the discrepancy between the entry criteria for the ADAPT trial and the indications for this treatment in real-world practice. Our results suggest future possibilities for efgartigimod treatment in patients with backgrounds different from those in the ADAPT trial. First, patients aged <18 years were excluded from the clinical trial (6). The inclusion criteria of the age for previous studies (12, 13) were similar to those of the ADAPT trial. Furthermore, studies in which the inclusion age was not described did not include patients aged <18 years (16). This study included one patient aged 15 years (Patient No. 3) who received efgartigimod. An open-label, phase 2/3 trial is ongoing to evaluate the pharmacokinetics, pharmacodynamics, efficacy, and safety of intravenous efgartigimod in children aged 2 to <18 years with generalized myasthenia gravis (23). In addition to the results of this trial, further studies on the use of efgartigimod for the treatment of gMG in patients aged <18 years are needed.

Second, patients who had experienced a myasthenic crisis (MGFA class V) during screening were excluded from the ADAPT trial. Unlike the clinical trials, previous studies included only a few patients with MGFA class V at baseline (13, 17). However, the outcomes of efgartigimod treatment in these patients have not been described in detail. In our study, five of 16 patients experienced myasthenic crisis at baseline, and four of these five patients were QMG responders in the first treatment cycle. In addition, as described in the clinical presentation of Patient No. 7, efgartigimod improved the QMG score of the patient with MGFA class V without rescue treatment. Watanabe et al. (24) reported a patient whose add-on treatment with efgartigimod resulted in recovery from refractory myasthenic crisis. We believe that efgartigimod may be useful in patients with gMG who have experienced myasthenic crisis. In general, standard care for managing a myasthenic crisis includes intravenous immunoglobulin and plasma exchange, which result in clinical improvement. Recent studies, such as the work by Vinciguerra et al., reported the initiation of eculizumab as a rescue therapy in refractory myasthenic crises (25, 26). We believe that efgartigimod may be a treatment option in patients with gMG who have experienced myasthenic crisis as well as other treatment including intravenous immunoglobulin, plasma exchange and eculizumab.

Third, patients were excluded from the clinical trials if they had received eculizumab or rituximab within the 6 months of screening. In our study, one patient received eculizumab and one patient received rituximab before the initiation of efgartigimod treatment. As described in the case presentation, Patient No. 4, who did not improve with eculizumab, showed clinically meaningful improvements in the QMG score after switching from eculizumab to efgartigimod. There are a few reports of switching from eculizumab or rituximab to efgartigimod (13–15, 17). Katyal et al. (13) reported two out of four patients who were on prior eculizumab therapy had clinically meaningful improvements in their MG-ADL scores after the administration of efgartigimod. In the study of Suzuki et al. (17), among the four patients who changed from eculizumab to efgartigimod, one patient showed remarkable effect of efgartigimod and among two patients who changed from rituximab to efgartigimod, one patient was responder (defined as having ≥2 point reductions in MG-ADL score). According to the recommendations of German guidelines, efgartigimod is an add-on therapy for highly active gMG with AChR antibodies (27). However, efgartigimod has not been mentioned in Japanese guidelines (17) because this drug was recently approved for the treatment of gMG in Japan. According to the Japanese guidelines for gMG, eculizumab or rituximab is recommended for refractory gMG with AChR antibodies (19). Patients with refractory gMG who do not respond to eculizumab or rituximab may have an option of receiving efgartigimod.

Our results may also support the use of efgartigimod as a steroid-sparing agent. In this study, we observed that patients could reduce their prednisolone dose after treatment with efgartigimod. Although the use of efgartigimod as a steroid-sparing agent was not investigated in the ADAPT trial, some groups reported that treatment with efgartigimod was able to reduce the daily dose of steroids (12, 14, 16, 17). The therapeutic goal in MG is to achieve maximal clinical benefit with a minimal dose of prednisolone, because a lower immunosuppressant dose is associated with a lower risk of adverse events (28). Further studies are required to evaluate the efficacy of efgartigimod as a steroid-sparing agent.

Adverse events observed in this study were similar to those in the ADAPT trial, which showed that efgartigimod was relatively safe and tolerable (6). In our study, the most common adverse events were headaches and diarrhea. Headache and diarrhea were observed in three patients (18.8%) and two patients (12.5%), respectively. In the clinical trial, the most common adverse events were headache, nasopharyngitis, nausea, diarrhea, upper respiratory tract infections, and urinary tract infections. Headache and diarrhea were reported in 24 patients (29%) and six patients (7%), respectively. Headache in our study was less than the clinical trial and diarrhea in this study was more than the clinical trial. No fatal or serious adverse events occurred in either of these studies. Unlike the clinical trial, we described a patient who developed a rash leading to treatment discontinuation after two infusions in the first cycle of efgartigimod. We should be cautious of rash during the administration of efgartigimod, although this may be uncommon.

Our study had a few limitations. We retrospectively analyzed data from patients with gMG who did not have a common background such as the treatment prior to the use of the efgartigimod. Since patients received several drugs prior to the use of the efgartigimod, the improvement of the patients in this study might be due to the efgartigimod alone as well as the efgartigimod plus other treatment like steroids. In addition, there was a small sample size and a lack of a control group in this study. This is why the generalizability of the findings is limited. Therefore, our findings need to be confirmed in larger prospective controlled studies.

**Figure 5:**
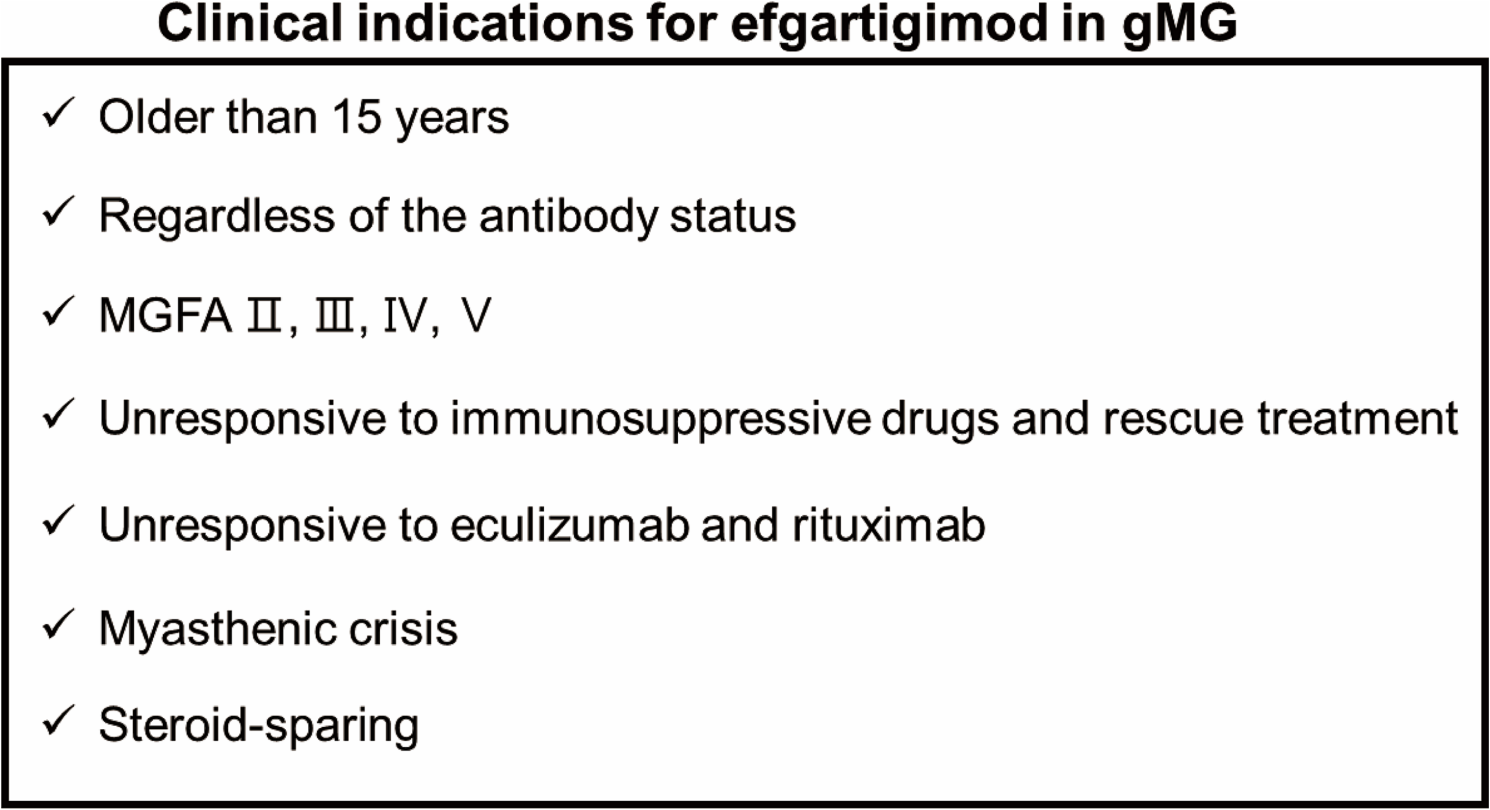
Clinical indications for efgartigimod in gMG.

In conclusion, we described our clinical experience with efgartigimod treatment in 16 patients with gMG from various backgrounds. In our study, the clinical outcomes and adverse events during the first cycle of efgartigimod treatment were consistent with those of the phase 3 ADAPT trial (6). Clinically meaningful improvements were observed in 14 patients, including one patient with myasthenic crisis and in one refractory patient who failed anti-complementary therapy. We also observed patients in whom the steroid dose was reduced after efgartigimod treatment. Clinical indications may be considered when treating gMG with efgartigimod as shown in Figure 5. Our experience suggests the future possibilities for the treatment of gMG with efgartigimod. In the future, our findings need to be validated in larger prospective clinical studies.

## Data Availability

The original contributions presented in the study are included in the article/supplementary material, further inquiries can be directed to the corresponding author.
